# Supplementary material for: Empirical methods for controlling false positives and estimating confidence in ChIP-Seq peaks
Source: BMC Bioinformatics. 2008 Dec 5;9:523. doi: 10.1186/1471-2105-9-523 (PMC2628906; doi:10.1186/1471-2105-9-523)
Supplement: Additional File 1 — A variety of html documents from the USeq web site detailing the available applications, their best usage, output file type descriptions, command line menus, etc. [file 1471-2105-9-523-S1.zip › USeqUserGuides/JavaDoc.zip!/JavaDoc/trans/roc/ControlGene.html]

ControlGene


|  |  |  |
| --- | --- | --- |
| **Overview** \| **Package** \| **Class** \| **Use** \| **Tree** \| **Deprecated** \| **Index** \| **Help** | |  |
| **PREV CLASS**   **NEXT CLASS** | **FRAMES**    **NO FRAMES**     **All Classes** |  |
| SUMMARY: NESTED \| FIELD \| CONSTR \| METHOD | DETAIL: FIELD \| CONSTR \| METHOD |  |


---


## trans.roc Class ControlGene

```
java.lang.Object
  trans.roc.ControlGene
```

---

``` public class ControlGene extends java.lang.Object ```

---

**Constructor Summary**

`ControlGene(java.lang.String line)`


| **Method Summary** | |
| --- | --- |
| `boolean` | `containsPoint(int bp)`             Looks to see if point is contained in any of the exons, ends are inclusive. |
| `java.lang.String` | `getChromosome()` |
| `int[]` | `getExonEnds()` |
| `int[]` | `getExonStarts()` |
| `float[]` | `getIntensities()` |
| `java.lang.String` | `getName()` |
| `java.lang.String` | `getNotes()` |
| `java.lang.String` | `getStrand()` |
| `static void` | `makeExons(ControlGene[] cgs, int endOffSet)`             Fires makeExons on each CG within the ControlGene[]. |
| `void` | `makeExons(int endOffSet)`             Subtracts the endOffSet from the end and only creates an exon if 1+ end-start > 0 |
| `static ControlGene[]` | `parseControlGeneFile(java.io.File file)`             parses a control gene file ex: #name chrom strand exonStarts exonEnds notes ex: NM\_001035267 chr12 + 54796640,54797239,54797532, 54796850,54797262,54797882, Z12962\_Homologue to yeast ribosomal protein L41\_H |
| `void` | `setIntensities(float[] intensities)` |
| `void` | `setNotes(java.lang.String notes)` |

**Methods inherited from class java.lang.Object**

`equals, getClass, hashCode, notify, notifyAll, toString, wait, wait, wait`

**Constructor Detail**

### ControlGene

```
public ControlGene(java.lang.String line)
```


**Method Detail**

### containsPoint

```
public boolean containsPoint(int bp)
```

:   Looks to see if point is contained in any of the exons, ends are inclusive.

---


### makeExons

```
public void makeExons(int endOffSet)
```

:   Subtracts the endOffSet from the end and only creates an exon if 1+ end-start > 0

---


### makeExons

```
public static void makeExons(ControlGene[] cgs,
                             int endOffSet)
```

:   Fires makeExons on each CG within the ControlGene[].

---


### parseControlGeneFile

```
public static ControlGene[] parseControlGeneFile(java.io.File file)
```

:   parses a control gene file
    ex: #name chrom strand exonStarts exonEnds notes
    ex: NM\_001035267 chr12 + 54796640,54797239,54797532, 54796850,54797262,54797882, Z12962\_Homologue to yeast ribosomal protein L41\_H

---


### getIntensities

```
public float[] getIntensities()
```

---


### setIntensities

```
public void setIntensities(float[] intensities)
```

---


### getNotes

```
public java.lang.String getNotes()
```

---


### setNotes

```
public void setNotes(java.lang.String notes)
```

---


### getChromosome

```
public java.lang.String getChromosome()
```

---


### getExonEnds

```
public int[] getExonEnds()
```

---


### getExonStarts

```
public int[] getExonStarts()
```

---


### getName

```
public java.lang.String getName()
```

---


### getStrand

```
public java.lang.String getStrand()
```


---


|  |  |  |
| --- | --- | --- |
| **Overview** \| **Package** \| **Class** \| **Use** \| **Tree** \| **Deprecated** \| **Index** \| **Help** | |  |
| **PREV CLASS**   **NEXT CLASS** | **FRAMES**    **NO FRAMES**     **All Classes** |  |
| SUMMARY: NESTED \| FIELD \| CONSTR \| METHOD | DETAIL: FIELD \| CONSTR \| METHOD |  |


---
